# Algal amendment enhances biogenic methane production from coals of different thermal maturity

**DOI:** 10.3389/fmicb.2023.1097500

**Published:** 2023-03-10

**Authors:** George A. Platt, Katherine J. Davis, Hannah D. Schweitzer, Heidi J. Smith, Matthew W. Fields, Elliott P. Barnhart, Robin Gerlach

**Affiliations:** ^1^Center for Biofilm Engineering, Montana State University-Bozeman, Bozeman, MT, United States; ^2^Department of Chemical and Biological Engineering, Montana State University-Bozeman, Bozeman, MT, United States; ^3^Department of Microbiology and Immunology, Montana State University-Bozeman, Bozeman, MT, United States; ^4^Department of Arctic and Marine Biology, Faculty of Biosciences, Fisheries and Economics, UiT The Arctic University of Norway, Tromsø, Norway; ^5^United States Geological Survey, Montana Water Science Center, Helena, MT, United States

**Keywords:** coal rank, methanogenesis, coal, methane, algal amendment

## Abstract

The addition of small amounts of algal biomass to stimulate methane production in coal seams is a promising low carbon renewable coalbed methane enhancement technique. However, little is known about how the addition of algal biomass amendment affects methane production from coals of different thermal maturity. Here, we show that biogenic methane can be produced from five coals ranging in rank from lignite to low-volatile bituminous using a coal-derived microbial consortium in batch microcosms with and without algal amendment. The addition of 0.1 g/l algal biomass resulted in maximum methane production rates up to 37 days earlier and decreased the time required to reach maximum methane production by 17–19 days when compared to unamended, analogous microcosms. Cumulative methane production and methane production rate were generally highest in low rank, subbituminous coals, but no clear association between increasing vitrinite reflectance and decreasing methane production could be determined. Microbial community analysis revealed that archaeal populations were correlated with methane production rate (*p* = 0.01), vitrinite reflectance (*p* = 0.03), percent volatile matter (*p* = 0.03), and fixed carbon (*p* = 0.02), all of which are related to coal rank and composition. Sequences indicative of the acetoclastic methanogenic genus *Methanosaeta* dominated low rank coal microcosms. Amended treatments that had increased methane production relative to unamended analogs had high relative abundances of the hydrogenotrophic methanogenic genus *Methanobacterium* and the bacterial family Pseudomonadaceae. These results suggest that algal amendment may shift coal-derived microbial communities towards coal-degrading bacteria and CO_2_-reducing methanogens. These results have broad implications for understanding subsurface carbon cycling in coal beds and the adoption of low carbon renewable microbially enhanced coalbed methane techniques across a diverse range of coal geology.

## Introduction

As the world transitions away from high carbon emitting fossil fuels towards cleaner energy sources, coal reserves may no longer be mined to the current extent, presenting economic challenges for communities that have previously relied on this resource (Jakob et al., 2020). Coalbed methane (CBM), which originates from both thermogenic and biogenic processes in coal beds, has been developed as an energy resource in many countries worldwide (Ritter et al., 2015; Davis and Gerlach, 2018). Efficient utilization of CBM could stimulate economic growth in coal-dependent regions of the world and address growing concerns surrounding emissions by providing a lower emissions natural gas fuel instead of coal (Ladage et al., 2021). Biogenic CBM is produced from the activity of *in-situ* microbial communities that catalyze the conversion of coal to methane in shallow coal beds (Barnhart et al., 2013; Ritter et al., 2015; Barnhart et al., 2016; Park and Liang, 2016; Barnhart et al., 2017; Davis and Gerlach, 2018; Schweitzer et al., 2019). To increase the economic attractiveness of CBM production, Microbially Enhanced Coalbed Methane (MECBM) techniques, which involve the stimulation of biogenic methane production in existing CBM wells, continue to be researched in the laboratory and in the field. A promising MECBM technique under investigation is the addition of very small amounts of an organic amendment, such as yeast extract or algal biomass, to *in-situ* microbial communities to provide essential nutrients and additional substrates to enhance coal dependent methane production (Barnhart et al., 2017; Davis and Gerlach, 2018; Davis et al., 2018a; Barnhart et al., 2022). The addition of algal extract to stimulate biogenic CBM production is particularly interesting because it is a low carbon, renewable stimulation method that may also be used to sequester carbon and recycle water involved in the CBM production process (Barnhart et al., 2017; Huang et al., 2017).

Research investigating the addition of algal amendment to stimulate CBM has primarily focused on identifying shifts in *in-situ* microbial community composition and metabolism following the addition of algal amendment and investigating the effect of reservoir conditions, such as formation water geochemistry and depth, on CBM production and coal degradation (Formolo et al., 2008; Dahm et al., 2011; Barnhart et al., 2013; Schweitzer et al., 2019, 2022). However, the effectiveness of algal amendment-based MECBM techniques in coal basins with different coal characteristics remains unclear. One such characteristic is coal rank; coal rank describes the level of physical and chemical transformation of coal organic matter as a result of thermal maturation (Suárez-Ruiz and Ward, 2008; Ward and Suárez-Ruiz, 2008; O'Keefe et al., 2013; Flores, 2014). As organic matter is coalified, plant components with similar chemical composition transition into subunits called macerals. Upon burial and geothermal heating, each maceral of the three major maceral groups (inertinite, liptinite, and vitrinite) undergoes unique geochemical changes controlled by vegetation type, redox potential (E_h_), and pH that target specific organic chemistries (Sýkorová et al., 2005; Scott and Glasspool, 2007; Hower et al., 2009; O'Keefe et al., 2013). The four main coal ranks are lignite, subbituminous, bituminous, and anthracite (O'keefe et al., 2013), with lignite being considered the lowest rank and anthracite the highest. Although no physical coal parameter perfectly explains coal rank, vitrinite reflectance (R_o_), the percent of incident light that is reflected from vitrinite macerals, is commonly used as a measure of coal rank and thermal maturity as it does not depend on coal composition or mineral matter (Teichmüller, 1989; Fakoussa and Hofrichter, 1999; Strąpoć et al., 2011; O'keefe et al., 2013).

The effect of coal rank on CBM production has been investigated previously due to its broad implications on potential CBM reserves. It is commonly assumed that the bioavailability of the recalcitrant coal organic matter decreases as coal rank increases due to the loss of heteroatoms such as oxygen, sulfur, and nitrogen, along with the condensation of the aromatic lignin-derived structures to higher order polyaromatic sheets (Drobniak and Mastalerz, 2006; Strąpoć et al., 2011; Fallgren et al., 2013; Robbins et al., 2016). However, this claim has been contested by multiple studies (Wawrik et al., 2012; Fallgren et al., 2013). Fallgren et al. (2013) suggest that methane production increases with coal rank when enrichments were set up in microcosms using coal samples from Argonne National Laboratory’s Premium Coal Sample Program. In contrast, Wawrik et al. (2012) found no correlation between coal rank and methane production when enrichments were prepared using coal samples from the Upper Cretaceous Fruitland Coal Formation in the San Juan Basin. A meta-analysis of published biogenic methanogenesis rates supported an increase in methanogenesis as rank decreased (Strąpoć et al., 2011), and an enrichment study using 14 coals of different rank as the sole carbon source showed a significant increase in methane production as rank decreased due to an increase in low molecular weight acids desorbing from the coal and increased bioavailability (Robbins et al., 2016). These studies draw conflicting conclusions but were ascertained using different enrichment methods. As a result, there is still uncertainty regarding the correlation between rank and biogenic methane production, if one exists at all.

In addition to understanding how coal rank affects biogenic methane production, the feasibility of MECBM using algal amendment across diverse coal geologies may support commercial application across a wide range of coal basins. It has been shown that amending ^13^C isotopically labeled algal concentrate into flow reactors containing subbituminous coal, formation water, and inoculum from Powder River Basin coal seams enhances coal-to-methane conversion (Davis et al., 2019). However, to the authors’ knowledge, the addition of an algal amendment to stimulate methane production in higher rank, lower methane producing coals has yet to be evaluated. Stimulating methane production from higher ranked coals may allow additional coal reserves to be utilized while reducing the negative consequences associated with traditional extraction techniques. The study presented here aims to re-assess the relationship between coal rank and microbial methane production, and to investigate the feasibility of stimulating biogenic methane production from coal of varying rank *via* the addition of algal amendment.

## Methods

### Coal sample collection and processing

Coal samples were obtained from the Argonne National Laboratory’s Premium Coal Sample Program (Argonne, IL, United States). The location, rank, and collection date for each coal sample are shown in Table 1. The collection and processing methods for these coals are detailed in Vorres (1990). To summarize, the lignite sample was collected from the Beulah-Zap coal seam in the Williston Basin (North Dakota, United States). The seam was approximately 18 ft. (5.5 m) thick at the collection area, and collection was done by accumulating core samples spaced approximately 20 ft. (6.1 m) apart (Vorres, 1990). One high volatile bituminous coal sample was collected from the Lewiston-Stockton seam of the Kanawha formation about 20 miles (32.2 km) east of Charleston, West Virginia, while the other was obtained from the Pittsburgh #8 seam approximately 60 miles (96.6 km) south of Pittsburgh, Pennsylvania. Both collection sites had a seam thickness of 6 ft. (1.8 m; Vorres, 1990). The low volatile bituminous coal sample was collected from the Pocahontas #3 seam in Buchanan County, Virginia, and the seam thickness was 6 ft. (1.8 m). The subbituminous B coal sample was collected from the Wyodak-Anderson seam approximately 6 miles (9.7 km) northeast of Gillette, WY. The seam was approximately 120 ft. (36.6 m) thick at the collection site and the sample used for processing consisted of a 6″ (15.3 cm) core spanning the thickness of the seam (Vorres, 1990). In addition to the Argon samples, a subbituminous C coal sample was collected from the Flowers-Goodale (FG) seam in the Powder River Basin in Rosebud County near Birney, Montana. Core samples from a well designated FGP-13 were extruded into a wooden trough and cleaned to remove drilling fluids. Samples were transferred to a disposable glove bag filled with N_2_ where the exposed outer core was removed (Barnhart et al., 2016). Proximate and ultimate analyses of the coal samples from the Argonne National Laboratory’s Premium Coal Sample Program (Vorres, 1990) and the Flowers-Goodale subbituminous C sample can be found in Table 2. FG proximate and ultimate analysis was obtained from Barnhart et al. (2016). All analyses were determined on a dry weight basis, except for percent hydrogen, which is reported on a dry, mineral-matter free basis, and heating value, which is reported on an as-received basis.

**Table 1:** Coal samples of different ranks used in this study.

Sample ID	Coal source	State	ASTM rank	Collection year
Lignite	Beulah-Zap	North Dakota	Lignite	1986
SubC	Flowers-Goodale	Montana	Subbituminous C	2013
SubB	Wyodak Anderson	Wyoming	Subbituminous B	1985
HV bit (Pittsburgh)	Pittsburgh #8	Pennsylvania	High Volatile Bituminous	1986
HV bit (Stockton)	Lewiston-Stockton	West Virginia	High Volatile Bituminous	1986
LV bit	Pocahontas #3	Virginia	Low Volatile Bituminous	1986

Sample locations and collection dates from Vorres (1990).

**Table 2:** Elemental and proximate analyses of each coal sample.

Sample ID	Ash (%)	Sulfur (%)	Fixed carbon (%)	Hydrogen (%)	R_o_ (%)	VM (%)	Heating value (BTU/lb)
Lignite	10.0	0.80	45.3	4.9	0.25	44.9	7,454
SubC	7.4	0.26	53.6	6.6	0.40	39.2	8,703
SubB	5.3	0.63	45.9	5.4	0.32	44.7	8,426
HV bit (Pittsburgh)	9.0	2.19	59.4	5.4	0.81	37.8	13,404
HV bit (Stockton)	20.0	0.71	53.5	5.4	0.89	30.2	11,524
LV bit	5.0	0.66	76.6	4.5	1.68	18.6	14,926

The subbituminous C (SubC) data for ash, sulfur, hydrogen, heating value, and volatile matter were obtained from the supplementary information in Barnhart et al. (2016).

### Site and sample collection

All water and microbial samples were collected from the Birney Test Site, previously described by Barnhart et al. (2016), located near Birney (MT) in the Powder River Basin (PRB). Formation water from the FG coal bed was obtained in June 2016. Water from the FG-09 well was collected in 6-gal (22.7 l) plastic jugs after pumping two well volumes and rinsing the jugs twice with formation water before filling and storing them at 4°C until microcosm set up. Coal cores were collected during the July 2013 drilling of the FG monitoring (FGM-13) and FG pumping (FGP-13) wells. The 2-inch (5.1 cm) diameter cores were cut into approximately 12-inch long (0.3 m) sections and placed in sealed PVC tubes filled with formation water pumped from the FG-11 well, which accesses the same FG coal source and aquifer. The tubes were sealed with flexible rubber caps to allow room for gas desorption. Microbial cultures were collected from the SS-13 well, which accesses the interface between the FG coal and the overlying sandstone, in September 2015 using the down-well samplers described by Barnhart et al. (2013). The microbial cultures (slurries) from the SS-13 sampler were added to a sterile serum bottle prepared with 5 g FG coal and 45 ml of filter sterilized, reduced formation water. The serum bottle was incubated at 21 ± 1°C in the dark for 361 days prior to being used as inoculum for the microcosms described below.

### Establishment of treatment cultures

Coal samples were received from the Argonne National Laboratory’s Premium Coal Sample Program in glass ampules. The glass ampules and the FG coal core (depth 384–385′) were opened in an anaerobic chamber (less than 5% H_2_ and less than 5% CO_2_), where it was dried and crushed. Crushed coal was sieved to an effective size of less than 850 μm. Prior to use in the microcosms, all coal samples were exposed to UV sterilization in a biosafety cabinet; during UV sterilization, the coal samples were briefly exposed to oxygen. Treatments were prepared in an anaerobic chamber by adding 1 g of each coal sample to autoclaved Balch tubes. To obtain a final liquid culture volume of 10 ml, each microcosm received 1 ml of previously collected enrichment culture described above and a balance of prepared formation water (9 ml for unamended treatments and 8 ml for amended treatments). Formation water was filtered (0.2-μm bottle top filters), sparged overnight with anoxic 5% CO_2_ (balance of N_2_) and treated with 1 mM sulfide (Na_2_S^●^9H_2_O) to remove residual oxygen. Resazurin was added to the reduced formation water to a concentration of 1 mM as a redox indicator. Microcosms were prepared in an anaerobic chamber by adding 1 g of each coal sample to autoclaved Balch tubes. To obtain a final liquid culture volume of 10 ml, each microcosm received 1 ml of previously collected enrichment culture described above and a balance of prepared formation water (9 ml for unamended treatments and 8 ml for amended treatments). One ml of an amendment suspension was added as appropriate. The amendment, a dried and crushed microalgal concentrate (*Chlorella* sp. strain SLA-04), was added to a final concentration of 0.1 g/l, as previously described (Davis et al., 2018b). Other than the microalgal concentrate, the microcosms received no other amendment. The Balch tubes were sealed with autoclaved butyl rubber stoppers and aluminum crimp seals before being removed from the anaerobic chamber. After removal, the headspace of the Balch tubes was replaced with a 5% CO_2_ gas mixture (balance of N_2_). Amended and unamended microcosms containing 1 mm borosilicate glass beads (GB) were set up as coal-free substrate controls. For each treatment (coal and glass beads), triplicate treatments were prepared. Treatments were incubated in the dark at room temperature (21 ± 1°C) for the duration of the 116-day experiment.

### Headspace gas measurements and analysis

Headspace gases (CH_4_ and CO_2_) were analyzed using an SRI Instruments (Torrance, CA, United States) Model 8,601 Gas Chromatograph (GC) equipped with a thermal conductivity detector (TCD) interfaced with PeakSimple Chromatography software. Ultra-high purity helium carrier gas and a Supelco HayeSep-D packed stainless-steel column (6 feet × 1/8” O.D) were used for separation. One ml of headspace gas was sampled from each microcosm and manually injected; the carrier gas had a pressure of 8 psi and the oven and TCD temperatures were 40°C and 150°C, respectively. To prevent creating a negative pressure in the tubes, 1 ml of anoxic 5% CO_2_ (balance N_2_) was injected to replace the sample volume removed. Reactors were sampled approximately every 2 weeks for the duration of the 116-day experiment.

### Microbial community analysis

On day 116, one replicate of each treatment was destructively sampled for DNA analysis, according to the protocol described in Davis et al. (2018b). Briefly, the coal or glass beads (GBs) and liquid fractions were separated by decanting the liquid fraction into a 15-ml Falcon conical centrifuge tube. The coal or GBs were transferred to separate 15-ml Falcon tubes. One ml of filter sterilized, 10% sodium dodecyl sulfate (SDS) was added to each gram of coal or GBs, and tubes were placed in a 70°C water bath for 30 min. The previously transferred liquid fraction in Falcon tubes was centrifuged, the supernatant decanted and discarded to leave approximately 2 ml with the resulting pellet. Both sample fractions were stored at -80°C until extraction. Just prior to DNA extraction, the coal/SDS mixture was heated in a 70°C water bath for an additional 30 min. Sample DNA was extracted using the FastDNA Spin Kit for Soil (MP Biomedical, Solon, OH) according to the manufacturer’s instructions with minor protocol changes, as reported in Davis et al. (2018b). After extraction, the DNA was prepared for polymerase chain reaction (PCR) amplification using the OneStep^™^ PCR Clean Up (Zymo Research, Irvine, CA). Extracted DNA was quantified using a Qubit fluorometer using the dsDNA HS Assay Kit (Thermo Fischer Scientific, Carlsbad, CA, United States). The 16S rRNA genes were PCR-amplified for 30 cycles with DreamTaq PCR Master Mix (Thermo Fischer Scientific, Carlsbad, CA, United States) and an annealing temperature of 55°C for 30 s using the universal prokaryotic primers Pro341F (5’-CCTACGGGNBGCASCAG-3′) and Pro805R (5’GACTACNVGGGTATCTAATCC-3′), which amplify the V4 region of the 16S rRNA genes of bacteria (Takahashi et al., 2014). Additionally, the archaea specific primers 751F (− CCGACGGTGAGRGRYGAA) and 1204R (− TTMGGGGCATRCIKACCT) were used to amplify the 16S rRNA gene of archaea (Baker et al., 2003).

Amplicons were checked by agarose gel electrophoresis with GelRed DNA stains (Biotium, Freemont, CA, United States). Library preparation for Illumina MiSeq was carried out following Illumina’s standard protocol “16S Metagenonic Sequencing Library Preparation” prior to being loaded for sequencing on the MiSeq v.3 platform (San Diego, CA, United States). To summarize, following PCR cleanup, purification, and indexing, DNA concentration was determined using the PicoGreen stain assay (Quant-IT, Invitrogen, Carlsbad, CA, United States). DNA concentrations were normalized and pooled with a 12.5% PhiX control library. Sequence reads were analyzed using the MiSeq standard operating procedure of the Mothur software package (Kozich et al., 2013). Forward and reverse reads were joined into contigs using QIIME (Caporaso et al., 2010). The sequences were aligned using SILVA (Quast et al., 2012). The aligned reads were quality filtered and chimeras were removed before OTUs and phylotypes were classified with an 80% confidence using the RDP database (Wang et al., 2007; Haas et al., 2011). Mothur version 1.38.1 was used to calculate species richness using Inverse Simpson Index (Supplementary Tables S1, S2).

### Statistical analysis

A Generalized Linear Model was fitted to the total amount of methane produced, maximum methane production rate, total carbon dioxide production, and carbon dioxide production rate with a factor for coal sample (rank) and a factor for the presence or absence of algal amendment using the statistical software Minitab v.18 (Minitab LLC, State College, PA). A value of p of less than 0.05 was used to determine statistical significance for all statistical analyses. Microbial community statistical analyses were performed in R v3.5.3 (R Development Core Team, 2019). Heatmaps of bacterial and archaeal relative abundances of combined OTUs based on phylotype at a minimum of 2.5% in at least one sample were generated using the CRAN packages Heatplus (Ploner, 2022), Vegan (Jari Oksanen et al., 2013), RColorBrewer (Neuwirth, 2022), and ggplots (Wickham, 2016). Principal Coordinate Analyses (PCoA) using Bray–Curtis Dissimilarity distances and non-transformed, combined OTU (based on phylotype) relative abundances (>2.5%) were used to explore differences in bacterial and archaeal community composition between microcosms. The environmental fitting function found in the CRAN package vegan was used to correlate community composition and coal sample metadata.

## Results

### Methane production

**Figure 1:**
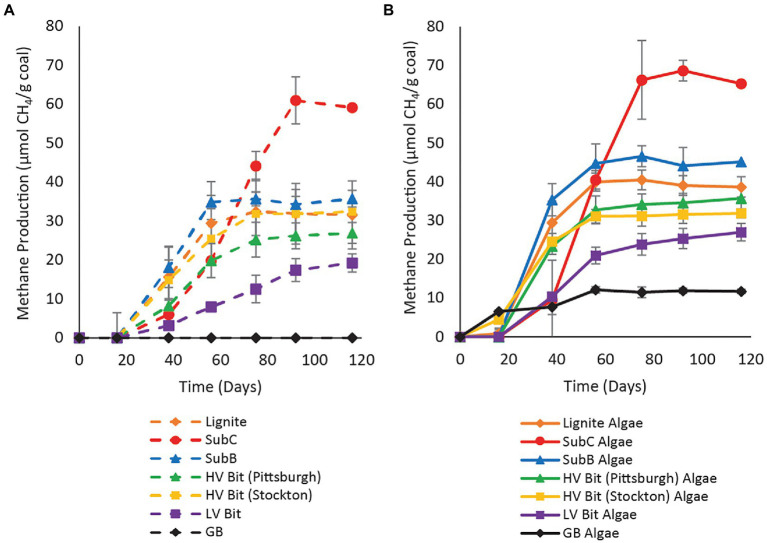
Methane production versus time for **(A)** unamended enrichment and **(B)** SLA-04 algae extract amended treatments. Algae extract was added to a final concentration of 0.1 g/l. Error bars represent one standard deviation of measurements from triplicate microcosms and are smaller than the markers where not visible.

Methane production was observed in all unamended microcosms, except for the negative control treatment containing glass beads instead of coal (Figure 1A), suggesting that coal-to-methane conversion occurred in all coal samples. Methane production was first detected on day 38 in all unamended coal treatments. The duration of methane production varied among microcosms, with Lignite producing methane until day 75, SubC until day 92, SubB until day 56, HV Bit (Pittsburgh) until day 75, HV Bit (Stockton) producing until day 75, and LV Bit until day 116. At the time of destructive sampling, cumulative methane production differed among microcosms. The unamended Lignite, SubC, SubB, HV Bit (Pittsburgh), HV Bit (Stockton), LV Bit, and GB microcosms produced 31.6 ± 8.8 μmol CH_4_/g coal, 59.2 ± 6.0 μmol CH_4_/g coal, 35.7 ± 2.1 μmol CH_4_/g coal, 26.9 ± 2.7 μmol CH_4_/g coal, 32.4 ± 6.2 μmol CH_4_/g coal, 19.3 ± 2.3 μmol CH_4_/g coal, and 0.0 ± 0.0 μmol CH_4_/g coal, respectively.

In algae-amended treatments, methane was detected earlier than in unamended treatments in Lignite Algae and GB Algae (methane was detected on day 16 already). The remaining amended microcosms showed detectable methane production by day 38 (Figure 1B) but at higher concentrations than the unamended treatments (*cf.* Figures 1A,B). The duration of methane production in algae amended treatments varied. For the Lignite Algae, SubC Algae, SubB Algae, HV Bit (Pittsburgh) Algae, HV Bit (Stockton) Algae, LV Bit Algae, and GB Algae, methane production appeared to cease on day 56, 75, 56, 56, 56, 116, and 56, respectively. At the end of the 116-day study, Lignite Algae, SubC Algae, SubB Algae, HV Bit (Pittsburgh), HV Bit (Stockton), LV Bit Algae, and GB Algae had produced 38.7 ± 2.6 μmol CH_4_/g coal, 65.3 ± 2.7 μmol CH_4_/g coal, 45.1 ± 4.7 μmol CH_4_/g coal, 35.7 ± 3.1 μmol CH_4_/g coal, 31.8 ± 1.1 μmol CH_4_/g coal, CH_4_/g coal, 27.0 ± 2.2 μmol CH_4_/g coal, and 11.7 ± 0.5 μmol CH_4_/g coal, respectively.

**Figure 2:**
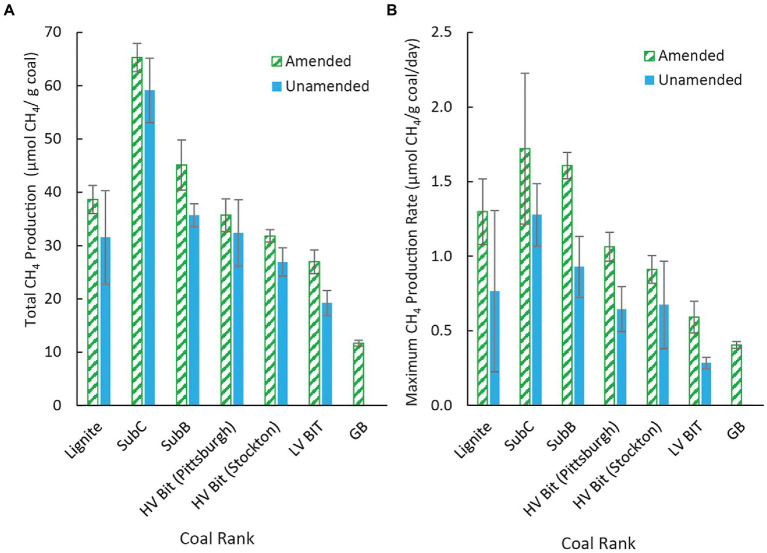
**(A)** Methane production in unamended microcosms and amended microcosms for coals of different rank and **(B)** Maximum methane production rate by coal rank for both amended and unamended microcosms. Maximum methane production rates occurred during different time intervals (see text), depending on the enrichment. Error bars represent one standard deviation of measurements from triplicate microcosms.

All unamended coal microcosms except for the unamended LV Bit enrichment (*p* = 0.56) produced significantly more methane than the amended and unamended GB (*p* < 0.05), indicating that the microbial community was able to convert some fraction of the coal substrate to methane. Methane was not observed in the unamended GB treatment, indicating that methane production from dissolved organics in the inoculum was negligible and that the source of methane in coal- or amendment-containing treatments was from coal or algal amendment. Additionally, coal rank had a statistically significant effect on total methane production in both unamended and amended microcosms (*p* < 0.05), although there was not an obvious statistical trend as vitrinite reflectance increased or decreased. For example, the cumulative methane production from the lowest ranked coal evaluated in this study (Lignite, R_O_ = 0.25%) grouped statistically with the methane production from the second highest ranked coal [HV Bit (Stockton), R_O_ = 0.89%]. The amended and unamended subbituminous C samples (SubC Algae, SubC, R_O_ = 0.40%) produced the highest amount of methane and the unamended low volatile bituminous coal sample (LV Bit, R_O_ = 1.68%) produced the lowest amount of methane of all the coal treatments. The remaining microcosms grouped statistically within these bounds, but without a strong correlation with vitrinite reflectance. A complete table of Tukey pairwise comparisons of cumulative methane production between treatments can be found in the Supplementary Table S3. On aggregate, the presence of algal amendment generally resulted in an increase in total methane production (*p* < 0.05), suggesting that methane production can be stimulated in different ranks of coal. Controlling for coal sample, no pairwise comparison (amended vs. unamended) resulted in a statistically significant difference in total methane production (Figure 2). However, comparing amended high rank samples to unamended low rank samples, comparable methane production was achieved. For example, methane production in the amended low volatile bituminous sample (LV Bit Algae) grouped statistically with the unamended subbituminous B (SubB) microcosm and both unamended high volatile bituminous microcosms (HV Bit (Stockton) and HV Bit (Pittsburgh)). This result suggests that the biogenic methane production potential of higher rank coals can be increased to be competitive with lower rank coals by the addition of small amounts of algae or similar amendments.

**Figure 3:**
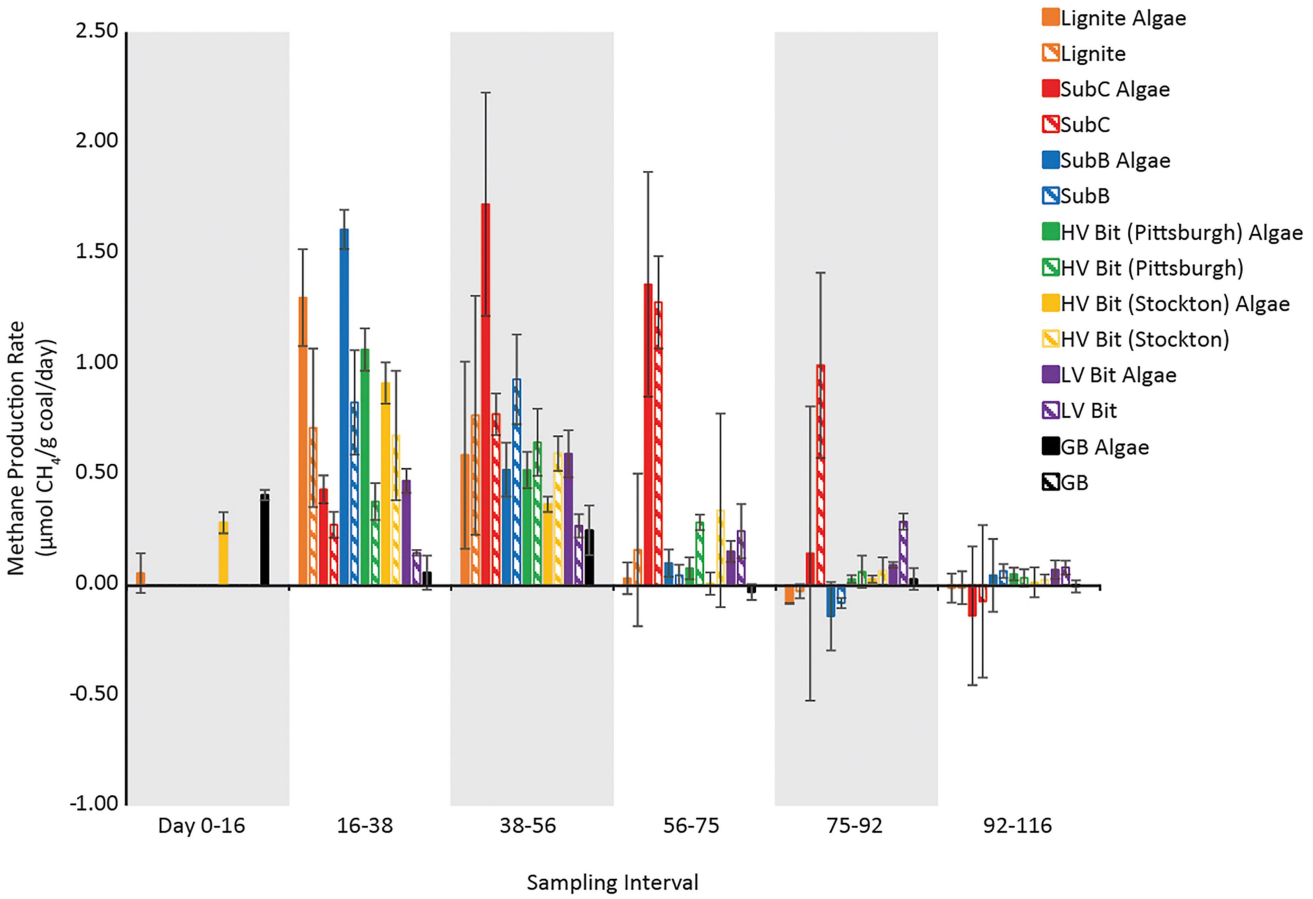
Methane production rates versus time for amended and unamended microcosms. Algae extract was added to a final concentration of 0.1 g/l. Error bars represent one standard deviation of measurements from triplicate microcosms.

Maximum methane production rates, and the time interval when they occurred, differed between coal microcosms. The maximum methane production rate for the unamended Lignite enrichment was 0.77 ± 0.54 μmol CH_4_/g coal/day between days 38 and 56 (Figure 3). The maximum methane production rate for the unamended SubC and SubB microcosms were 1.28 ± 0.21 μmol CH_4_/g coal/day between day 56 and 75 and 0.93 ± 0.20 μmol CH_4_/g coal/day between day 38 and 56, respectively (Figure 3). The maximum methane production rates for the unamended high volatile bituminous microcosms HV Bit (Pittsburgh) and HV Bit (Stockton) were 0.64 ± 0.15 μmol CH_4_/g coal/day between day 38 and 56 and 0.67 ± 0.29 μmol CH_4_/g coal/day between day 16 and 38, respectively. Lastly, the maximum methane production rate for the unamended low volatile bituminous enrichment, LV Bit, was 0.29 ± 0.04 μmol CH_4_/g coal/day between day 75 and 92. Coal rank had a statistically significant effect on maximum methane production rate (*p* < 0.05), with the highest rank coal microcosm (LV Bit, R_O_ = 1.68%) having the lowest methane production rate. However, a clear trend between increasing coal rank and methane production rate could not be determined.

The addition of algal amendment also had a statistically significant effect on methane production rate (*p* < 0.05), with algae-amended microcosms generally exhibiting greater methane production rates than unamended microcosms (Figure 3). The interaction between the effect of algal amendment on methane production rate and coal rank was not statistically significant (*p* = 0.78), suggesting that algal amendment can stimulate methane production regardless of coal rank. Complete methane production rate statistical analysis results and Tukey pairwise grouping can be found in the Supplementary Table S4. The maximum methane production rate for the amended Lignite Algae enrichment was 1.30 ± 0.22 μmol CH_4_/g coal/day, occurring between day 16 and 38. The maximum methane production rate for the amended SubC Algae and SubB Algae microcosms were 1.72 ± 0.51 μmol CH_4_/g coal/day and 1.61 ± 0.09 μmol CH_4_/g coal/day, occurring between day 38 and 56 and day 16 and 38, respectively. The amended high volatile bituminous treatments, HV Bit (Pittsburgh) and HV Bit (Stockton), had maximum methane production rates of 1.06 ± 0.10 μmol CH_4_/g coal/day between day 16 and 38 and 0.91 ± 0.09 μmol CH_4_/g coal/day between day 16 and 38. Lastly, the maximum methane production rates for the amended LV Bit and amended GB Algae treatments were 0.59 ± 0.11 μmol CH_4_/g coal/day and 0.41 ± 0.02 μmol CH_4_/g coal/day, respectively. Notably, amended treatments had earlier maximum methane production rates compared to corresponding unamended treatments, except for the HV Bit (Stockton) treatments, which had maximum methane production rates between the same sampling days (between day 16 and day 38). Maximum methane production rates in amended treatments were observed 18–37 days earlier compared to unamended treatments (Figure 3), and methane production leveled out 17–19 days earlier in Lignite Algae, SubC Algae, HV Bit (Pittsburgh) Algae and HV Bit (Stockton) Algae compared to analogous unamended treatments (Figure 1). These results suggest that the presence of the algal amendment decreased the lag time before the onset of methane production in these treatments. The SubB Algae and LV Bit Algae treatments ceased methane production approximately at the same time as the corresponding unamended treatments, but elevated maximum methane production rates were observed. These results suggest that while final methane production may not increase, the addition of algal amendment can decrease lag times before methane production commences and can increase production rates across different coal ranks.

### Carbon dioxide production and consumption

Depending on the methanogenic pathway, inorganic carbon (CO_2_) can be either a substrate or a product, and CO_2_ can therefore be an indicator of the type of methanogenesis occurring in a system. Thus, the mass of CO_2_ produced in the headspace of the microcosms was monitored for the duration of the experiment. Production of inorganic carbon in unamended microcosms was associated with coal rank (*p* < 0.05), with subbituminous coals generally producing more CO_2_ than bituminous coals. After accounting for CO_2_ added during sampling (through the replacement of headspace gas), unamended Lignite, SubC, SubB, HV Bit (Pittsburgh), HV Bit (Stockton), LV Bit, and GB microcosms produced 1.1 ± 1.7 μmol CO_2_/g coal, 72.0 ± 4.7 μmol CO_2_/g coal, 15.2 ± 2.6 μmol CO_2_/g coal, 6.0 ± 1.5 μmol CO_2_/g coal, 3.6 ± 5.6 μmol CO_2_/g coal, 3.7 ± 1.2 μmol CO_2_/g coal, and − 3.1 ± 2.1 μmol CO_2_/g coal, respectively by the end of the 116-day study (Supplementary Figure S1). A “negative” production in the GB treatment headspace suggests that CO_2_ was consumed. Because no methane was detected in this treatment, the consumption of CO_2_ is likely the result of CO_2_ dissolution into the aqueous phase and subsequent speciation into H_2_CO_3_, HCO_3_^−^, and CO_3_^2−^ as opposed to hydrogenotrophic methanogenesis; no photosynthesis is expected to have occurred in the microcosms because they were incubated in the dark. While subbituminous coals generally produced more CO_2_ than higher rank coals, the lignite treatment did not follow this trend, grouping more closely with the low volatile bituminous coal (LV Bit). An increased production of inorganic carbon in the lower rank coals may suggest the increased degradation of organic matter, which is supported by generally higher amounts of methane production in these treatments.

Amended Lignite Algae, SubC Algae, SubB Algae, HV Bit (Pittsburgh) Algae, HV Bit (Stockton) Algae, LV Bit Algae, and GB Algae microcosms produced 4.6 ± 0.3 μmol CO_2_/g coal, 75.8 ± 0.4 μmol CO_2_/g coal, 23.6 ± 3.5 μmol CO_2_/g coal, 12.8 ± 1.9 μmol CO_2_/g coal, 12.6 ± 2.7 μmol CO_2_/g coal, 2.6 ± 6.5 μmol CO_2_/g coal, and 7.8 ± 2.5 μmol CO_2_/g coal, respectively. Once again, amended subbituminous microcosms produced more CO_2_ than amended high volatile and low volatile bituminous samples. Consistent with the unamended lignite treatment, the amended lignite treatment did not group with the lower rank coals and instead produced similar amounts of CO_2_ as the higher rank bituminous coals. The effect of algal amendment on CO_2_ production was significant (*p* < 0.05), while the interaction between algal amendment and rank was not (*p* = 0.17), suggesting that the effect of algal amendment on CO_2_ production does not depend on the rank of the coal sample. Controlling for coal sample, there were no instances in which algae-amended treatments produced significantly more CO_2_ than analogous unamended treatments. A complete table of Tukey pairwise comparisons of cumulative CO_2_ production can be found in the Supplementary Table S5.

Maximum CO_2_ production rates (Supplementary Table S6) occurred between day 0–16 for the amended and unamended coal microcosms, except for the amended and unamended lignite sample, which had a maximum production rate between day 38 and 56, and the amended low volatile bituminous treatment (LV Bit Algae), which had a maximum production rate between day 56 and 75. Unamended Lignite, SubC, SubB, HV Bit (Pittsburgh), HV Bit (Stockton), LV Bit, and GB microcosms had maximum CO_2_ production rates of 0.2 ± 0.1 μmol CO_2_/g coal/day, 3.3 ± 0.2 μmol CO_2_/g coal/day, 0.7 ± 0.1 μmol CO_2_/g coal/day, 0.3 ± 0.1 μmol CO_2_/g coal/day, 0.2 ± 0.3 μmol CO_2_/g coal/day, 0.2 ± 0.1 μmol CO_2_/g coal/day, and − 0.4 ± 0.0 μmol CO_2_/g coal/day, respectively. Similarly, amended microcosms Lignite Algae, SubC Algae, SubB Algae, HV Bit (Pittsburgh) Algae, HV Bit (Stockton) Algae, LV Bit Algae, and GB Algae produced 0.2 ± 0.2 μmol CO_2_/g coal/day, 3.5 ± 0.0 μmol CO_2_/g coal/day, 1.1 ± 0.2 μmol CO_2_/g coal/day, 0.6 ± 0.1 μmol CO_2_/g coal/day, 0.6 ± 0.1 μmol CO_2_/g coal/day, 0.12 ± 0.0 μmol CO_2_/g coal/day, and 0.2 ± 0.0 μmol CO_2_/g coal/day, respectively. As with total CO_2_ production, the maximum production rate was generally higher in subbituminous coals than in bituminous coals (Supplementary Table S6).

Lignite treatments had maximum CO_2_ production rates that occurred 40 days later than subbituminous and high volatile bituminous treatments and were closer in magnitude to the low volatile bituminous treatments. Similar to maximum methane production rates, the effect of algae on maximum CO_2_ production rates was statistically significant (*p* < 0.05), with amended treatments on aggregate generally having greater CO_2_ production rates than unamended treatments. The effect of algae on maximum CO_2_ production rate was not significantly dependent on the rank of coal (*p* = 0.15), with the grouping of treatments generally mirroring total CO_2_ production and methane production. In both amended and unamended coal microcosms, maximum CO_2_ production rates occurred earlier than maximum methane production rates, except for the amended and unamended lignite treatments and the amended low volatile bituminous treatment.

### Microbial community analysis

#### Characterization of bacterial and archaeal community composition

**Figure 4:**
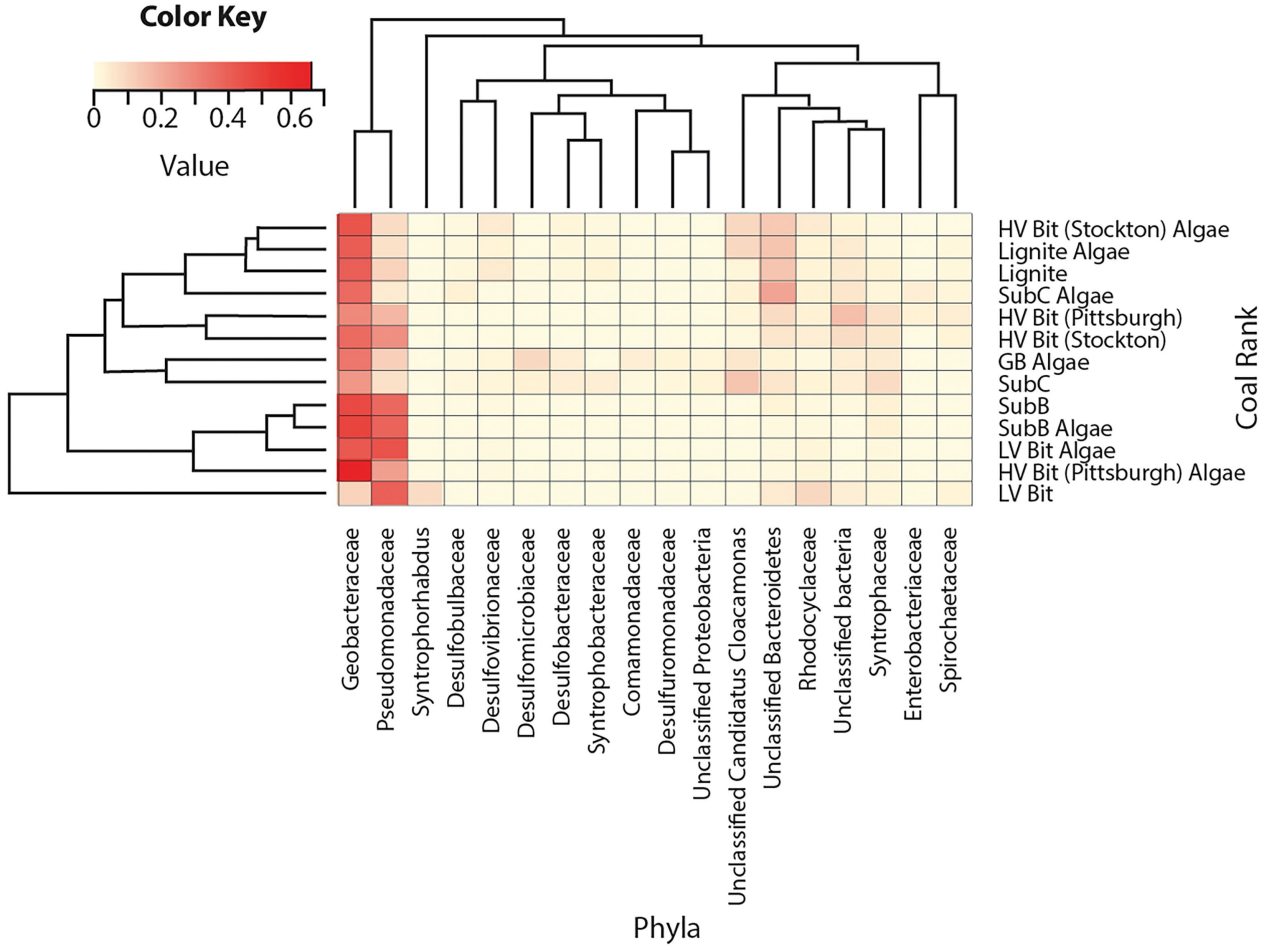
Heatmap of bacterial relative abundance of OTUs combined by phylotype. Phylotypes without a relative abundance of 2.5% in at least one sample were omitted.

Microbial community analysis revealed an average of 102 ± 42 observed bacterial operational taxonomic units (OTUs) and 144 ± 69 observed archaeal OTUs among all sequenced coal treatments. Archaeal DNA from the SubC Algae treatment and both bacterial and archaeal DNA from the GB treatment were not successfully amplified, therefore these treatments were omitted from the following analyses. Sequences indicative of species found in the bacterial family Geobacteraceae were found in all microcosms at relative abundances between 11 and 67% with no apparent dependency on rank or presence of algal amendment (Figure 4). Sequences classified within the bacterial family Pseudomonadaceae (5–46%) were present in all microcosms and were most abundant in both LV Bit and both SubB microcosms (39–46%) compared to the other microcosms (5–29%). These microcosms, in addition to HV Bit (Pittsburgh) Algae, had less than 0.01% abundance of unclassified species from the candidate phylum Cloacamonas, while other microcosms, such as SubC, had relative abundances as high as 15% for this phylum. Sequences indicative of species found in the Syntrophaceae family, previously associated with the degradation of crude oil alkanes (Gray et al., 2011) were present in all microcosms at low relative abundance (1–9%).

**Figure 5:**
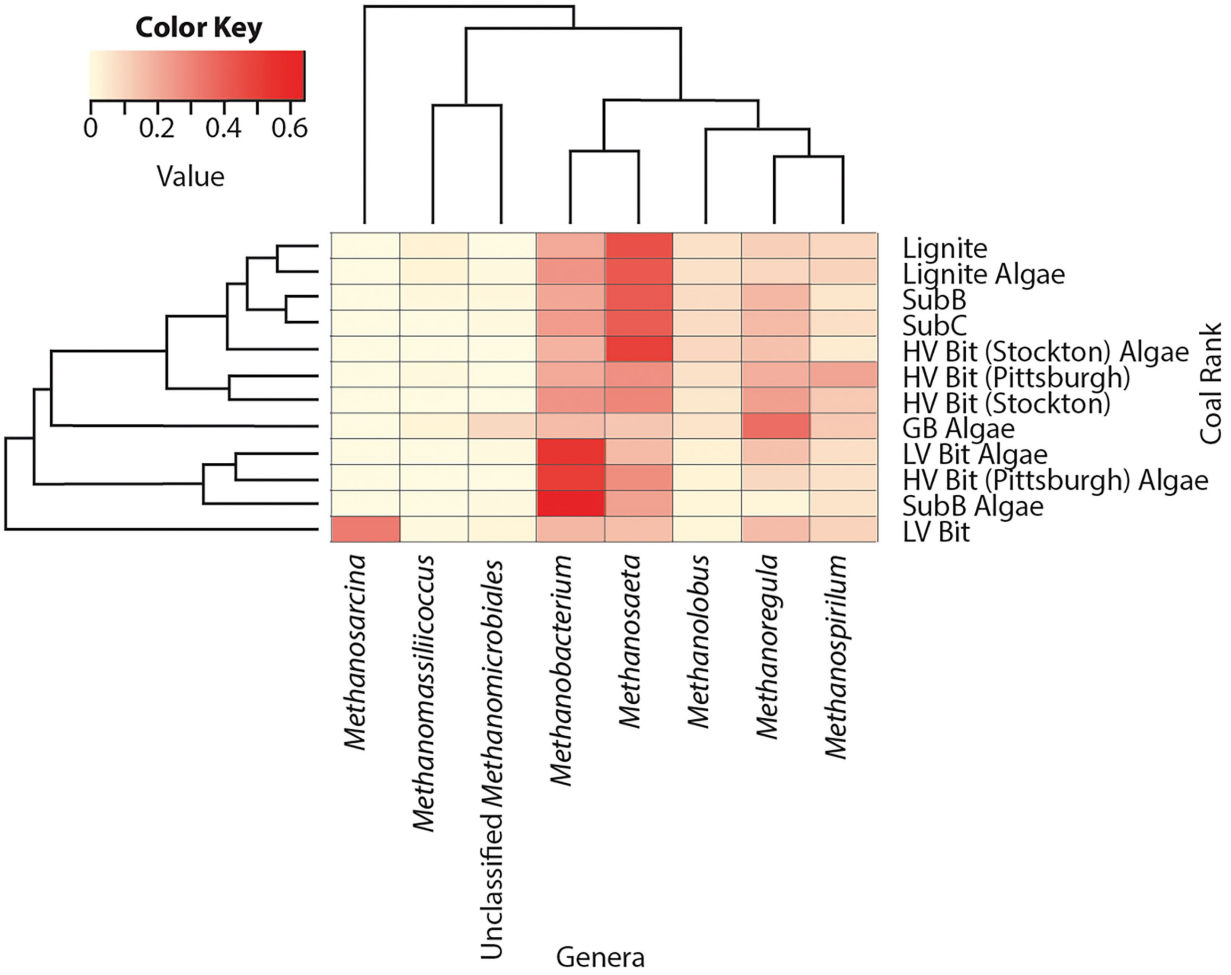
Heatmap of archaea relative abundance of OTUs combined by phylotype. Phylotypes without a relative abundance of 2.5% in at least one sample were omitted.

Sequences indicative of species in the archaeal genera *Methanobacterium* (16–65%), *Methanosaeta* (14–50%), *Methanoregula* (2–37%), *Methanolobus* (2–10%) and *Methanospirillum* (5–22%) were found in all sequenced microcosms, indicating relatively homogeneous archaeal populations in all microcosms (Figure 5). OTUs from the genus *Methanosaeta* had high relative abundances (41–45%) in low rank coal microcosms (Lignite, Lignite Algae, SubB, and SubC) and low relative abundances (16, 17%) in the highest rank microcosms (LV Bit, LV Bit Algae). The LV Bit Algae, HV Bit (Pittsburgh) Algae, and Sub B Algae microcosms all showed high relative abundances (52–65%) of *Methanobacterium*, a genus associated with hydrogenotrophic methanogenesis (Ünal et al., 2012) compared to other microcosms (16–26%). These microcosms also had lower relative abundances of *Methanosaeta* (17–28%), a group of methanogenic archaea that have been described to produce methane from acetate (Meslé et al., 2013). Members of the genus *Methanosarcina*, which have been shown to utilize all three known methanogenesis pathways (De Vrieze et al., 2012), were not found in any microcosms except LV Bit, which had a high relative abundance (28%) of this genus. The highest abundance of the genus *Methanoregula*, often associated with hydrogenotrophic methanogenesis (Gründger et al., 2015), was found in the GB Algae enrichment (37%).

#### Microbial community composition and coal properties

**Figure 6:**
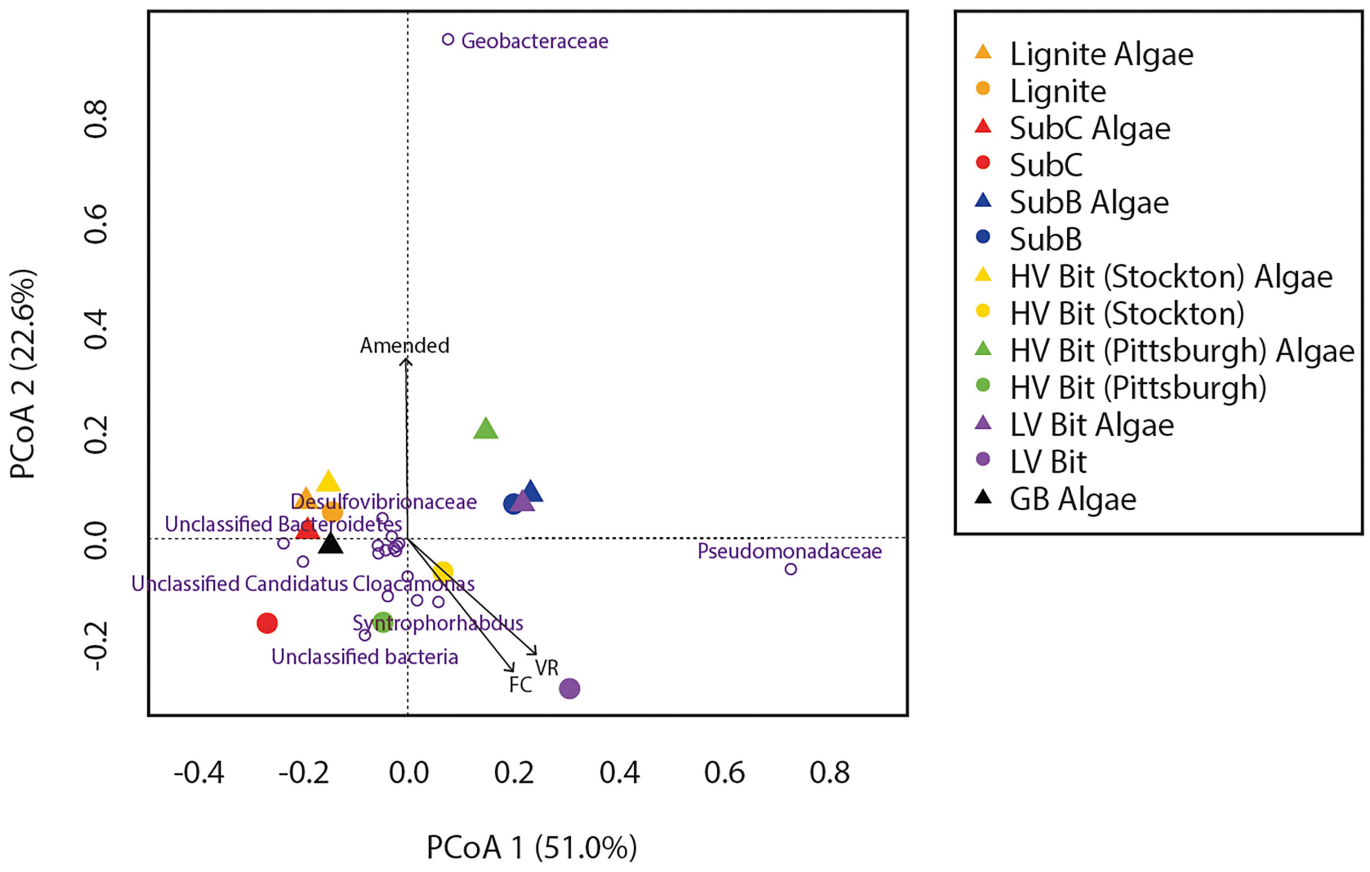
Principal Coordinate Analysis (PCoA) of non-transformed bacterial relative abundance based on OTUs combined by common phylotype with vectors representing significantly (or partially significant, *p* < 0.10) correlated coal composition and gas production parameters.

**Figure 7:**
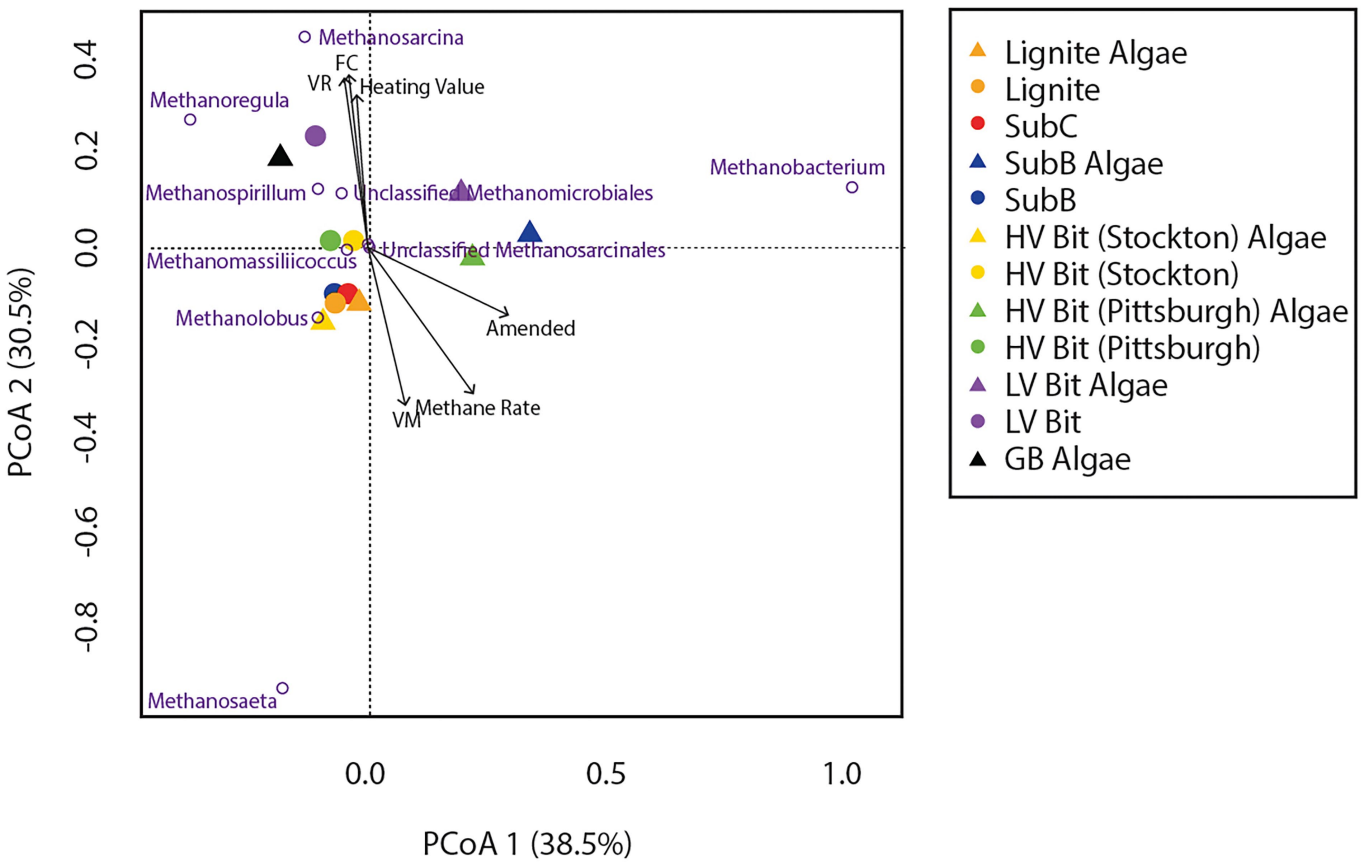
Principal Coordinate Analysis (PCoA) of non-transformed archaeal relative abundance based on OTUs combined by common phylotype with vectors representing significantly (or partially significant, *p* < 0.10) correlated coal composition and gas production parameters.

Principal Coordinate Analyses (PCoA) were used to elucidate differences in bacterial and archaeal community composition between amended and unamended microcosms grown on coals of different thermal maturity (Figures 6, 7). For the bacterial communities, no discernable clustering was apparent based on community composition alone. Based on the bacterial PCoA diagram (Figure 6), the amended SubC Algae, HV Bit (Stockton) Algae, HV Bit (Pittsburgh), and LV Bit Algae microcosms showed different community composition relative to their unamended analogs. A component of all community composition variation between amended and unamended treatments was along PCoA axis 2, which was correlated with OTUs from the family Geobacteraceae. The variation associated with the HV Bit (Stockton) Algae was also associated with variation along PCoA axis 1, which appeared to most closely correlate with unclassified OTUs from the phylum Bacteroidetes, the phylum Pseudomonadaceae, and the candidate phylum Cloacamonas. The variation associated with the HV Bit (Pittsburgh) Algae enrichment also contained a component in the positive PCoA axis 1 direction, associated with OTUs from the bacterial family Pseudomonadaceae. Environmental fitting with coal proximate and ultimate analysis and methane production revealed no significant correlations at the 95% confidence level. Weak correlations between bacterial community composition and the presence of algal amendment (Amended, r^2^ = 0.47, *p* = 0.06), vitrinite reflectance (R_o_, r^2^ = 0.42, *p* = 0.08), and fixed carbon (FC, r^2^ = 0.39, *p* = 0.10) were observed. These results suggest that bacterial community composition may be affected by coal properties and the addition of algal amendment, but demonstrably significant correlations could not be verified.

PCoA analysis for archaeal community composition (Figure 7) showed tight clustering of unamended microcosms except for the unamended low volatile bituminous treatment (LV Bit). Like the observed dissimilarities in bacterial community composition, algal amendment resulted in significant variations in archaeal community composition for the SubB, HV Bit (Pittsburgh), and LV Bit microcosms (Figure 7). This archaeal community shift appears to be associated with OTUs from the genus *Methanobacterium*, which has been correlated with hydrogenotrophic methanogenesis. Community composition for unamended microcosms was associated with OTUs from the genera *Methanolobus* and *Methanosaeta*, which have been described to generally be unable to reduce carbon dioxide in the presence of hydrogen and instead utilize methyl compounds (Munson et al., 1997) and acetate (Meslé et al., 2013), respectively. Significant correlations were revealed between archaeal community composition and percent volatile matter (VM, *r^2^* = 0.57, *p* = 0.05), fixed carbon (FC, *r*^2^ = 0.68, *p* = 0.02), vitrinite reflectance (VR, *r*^2^ = 0.65, *p* = 0.03), maximum methane production rate (*r*^2^ = 0.68, *p* = 0.01), the presence of algal amendment (Amended, *r*^2^ = 0.51, *p* = 0.04), and heating value (HV, *r*^2^ = 0.53, *p* = 0.05). Like the bacterial community composition, no correlation was found with total methane production, ash, sulfur, or hydrogen content of the coal. Significant correlations between archaeal community composition and coal properties suggest that coal composition and rank affect the availability of intermediate substrates used for methanogenesis. Due to the syntrophic nature of coal-to-methane conversion, available intermediates for methanogenesis are also dependent on the ability of hydrolytic and fermentative bacteria to produce them.

## Discussion

Determining the effectiveness of MECBM strategies across a range of coal thermal maturity may contribute to understanding commercially viability of the enhancement technique. While lower rank coals are typically targeted for CBM production, enhancing methane production in higher rank coals may allow increased utilization of in-ground resource and potentially the waste generated during mining and coal combustion pre-treatment. Microcosms amended with algae generally produced more methane, had higher methane production rates than unamended treatments, and had earlier maximum methane production rates relative to their unamended analogs. After amending with algae, the higher ranked coals, like HV Bit (Stockton) and HV Bit (Pittsburgh), showed comparable amounts of headspace methane to lower rank coals such as the SubB and Lignite that were unamended, suggesting that amended higher rank coals seams could be used as a concurrent source of methane in parallel with methane producing low rank coal seams. Previous studies have shown that adding algal amendment to coal microcosms stimulates methane production by enhancing coal degradation, likely by providing hydrolytic and fermentative bacteria with limiting nutrients (Barnhart et al., 2017, 2022; Davis and Gerlach, 2018; Davis et al., 2018a,b). In this study, increased methane production could not be explicitly shown to be from enhanced coal degradation alone based on carbon mass balance analyses, suggesting that the microbial consortium may have converted some amendment directly to methane. However, our previous studies, including those using ^13^C-labeled algal amendment, have shown that adding algal amendment to a final concentration of 0.1 g/l results in more carbon produced as methane than the amount of carbon that was added as amendment (Davis et al., 2018a,b), suggesting that direct amendment conversion cannot solely explain the increase in observed methane yields. Lastly, while microcosms amended with algae did not always exhibit an increase in total methane yield, they did show greater methane production rates and decreased lag times, which is important for commercial-scale applications and highlights the feasibility of stimulating methane production using an organic amendment across a range of coal ranks and thus coal basins.

This investigation confirms that coal-derived microbial communities can produce significant quantities of methane from many different ranks of coal, and that organisms capable of utilizing complex organic matter are involved in this process (Smith et al., 2021). It was found that coal rank has a significant effect on methane production, with subbituminous coals producing more methane than bituminous coals. These results are consistent with previous published studies (Strąpoć et al., 2011; Robbins et al., 2016), but contradict a study conducted by Fallgren et al. (2013), who used the same coal samples from the Argonne National Laboratory Premium Coal Sample Program. Robbins et al. (2016) investigated methane production in microcosms inoculated with biomass from a wide range of organic substrate-degrading organisms (termite guts, digester fluid, koala feces, lake sediment, and CBM production water) using 14 coals of different rank as the sole carbon source. When methane production leveled out after 50 days, Robbins et al. (2016) reported methane yields from 0.2–26.4 μmol CH_4_/g coal. These methane yields are comparable to the unamended yields observed in this study after 50 days (6.2–28.7 μmol CH_4_/g coal) but are generally lower than the yields observed in this study after 116 days (19.3–59.2 μmol CH_4_/g coal).

Compared to Fallgren et al. (2013), the methane yields in this study through 60 days were 71.7–99.2% higher (Supplementary Table S7) and showed a negative correlation between increasing coal rank and methane production, apart from the lignite sample. The increase in methane yield shown in this study, and the opposite correlation with coal rank, may highlight the importance of coal-derived organisms associated with the degradation of the coal geopolymer. Fallgren et al. (2013) also used coal samples from the Argonne National Laboratory Premium Coal Sample Program but used methanogenic enrichment cultures from coal formation water as opposed to the enrichment culture from a coal slurry as used in this study. There is evidence that the planktonic microbial community associated with coal formation water is not indicative of the coal-associated microbial community associated with coal slurry samples collected using a downhole sampler (Schweitzer et al., 2019). It is possible the discrepancy between methane yields from the same coal samples may have been due to an absence of coal degrading organisms in the formation water culture used by Fallgren et al. (2013). Our results suggest that the presence of a native, coal-derived microbial community may play a more influential role in coalbed methane production than coal rank, and that previous studies investigating the relationship between rank and methane production have likely underestimated the methane potential from higher rank coals. Moving forward, to more accurately predict *in-situ* methane production, coal-associated microbial communities could be used in laboratory studies, with care taken to mimic *in-situ* conditions.

Additionally, adsorption capacity is related to coal micropore structure development, which is dependent on coal rank and maceral composition (Meng et al., 2019). In this study, the lignite coal deviated from the proposed methane-rank correlation. This deviation could be attributed to preferential methane sorption over CO_2_ at low pressures (Busch et al., 2003; Busch and Gensterblum, 2011). Busch et al. (2003) showed that the Beulah-Zap lignite coal used in this study had an extraordinarily high methane adsorption capacity relative to other lignite coals, suggesting that the coal-to-methane conversion of this sample may have been consistent with expected methane production based on rank but that all methane produced was not detected due to high methane adsorption. In the low-pressure range, it is well documented that adsorption capacity increases with a decreasing amount of volatile matter and an increase in vitrinite reflectance, resulting in an increase in coal micro-porosity at higher rank (Crosdale et al., 1998; Prinz et al., 2001). Because of absolute and preferential CH_4_/CO_2_ adsorption changes with coal rank and maceral composition, the amount of biological methane produced from higher rank coals in laboratory studies may need re-evaluation. Additionally, it has also been reported that microbial conversion of coal affects coal pore structure (Zhang et al., 2017) and thus adsorption capacity, further complicating the issue. If the degree of microbial coal degradation is dependent on rank, so might changes in adsorption capacity.

Microbial community composition after 116 days of incubation was found to be correlated with coal composition and the coal proximate analysis results. Bacterial community composition was dominated by sequences indicative of species from the families Geobacteraceae and Pseudomonadaceae. These microbial communities have previously been identified in other *in-situ* CBM investigations although the results presented here are the first to indicate they can remain dominant across coal ranks (Shimizu et al., 2007; Fry et al., 2009; Barnhart et al., 2013; Singh and Tripathi, 2013; Schweitzer et al., 2019, 2022). Species from the family Geobacteraceae, well known for containing organisms capable of acetate and monoaromatic hydrocarbon oxidation, have previously been detected in coal mine deposits (Shimizu et al., 2007; Shin et al., 2008; Fry et al., 2009; Beckmann et al., 2011) and appear to play a role in the breakdown of complex organic matter. Members of the family Pseudomonadaceae, who are facultative anaerobes often capable of utilizing n-alkanes, polyaromatic hydrocarbons, and heterocyclic compounds, are often associated with the hydrolysis of coal geopolymer hydrocarbons into bioavailable intermediates (Deziel et al., 1996; Juhasz et al., 1996; Mittal and Rockne, 2008; Zhang et al., 2011). They are also well-known biosurfactant producers, which has been hypothesized to be an important enzymatic process involved in coal degradation (Deziel et al., 1996; Bognolo, 1999; Zheng et al., 2012; Singh and Tripathi, 2013; Kügler et al., 2015; Barnhart et al., 2016; Schweitzer et al., 2022). Although the mechanism of increased methane production could not be directly ascertained from observed methane yields and rates, the elevated abundance of members of the Pseudomonadaceae family in the most productive amended microcosms suggests that additional methane may be the result of enhanced coal hydrolysis rather than direct amendment conversion. This shift was not found in the GB Algae enrichment, further supporting the hypothesis that the presence of coal and algae may have selected for coal degrading organisms in some microcosms. Furthermore, the elevated abundance of members of the Pseudomonadaceae family and increased methane production rates in both subbituminous (lower rank) and low volatile bituminous (higher rank) coal treatments suggests that the addition of algal amendment can select for coal-degrading organisms, regardless of coal rank. This result supports the hypothesis that it is possible to enhance the degradation of coals with generally low bioavailability by amending them with small amounts of an easily degradable organic amendment, such as microalgal biomass (Smith et al., 2021).

The archaeal communities in both amended and unamended microcosms were composed mostly of organisms from the genera *Methanobacterium, Methanosaeta, Methanoregula, Methanolobus* and *Methanospirillum*. Amended microcosms that resulted in the largest increase in methane production were enriched for *Methanobacterium*, a mesophilic hydrogenotrophic methanogen (Ünal et al., 2012). Interestingly, the amended GB enrichment did not cluster with these treatments (Figure 7) and instead was associated with high relative abundance of *Methanoregula* (Smith et al., 2021), also associated with hydrogenotrophy. This result suggests that organisms involved in enhanced coal-associated methanogenesis are different from those who produce methane *via* direct amendment conversion. Organisms from the genus *Methanosaeta* were more abundant in the lower rank coal microcosms than higher rank microcosms, suggesting that the bioavailability of lower rank coal promotes acetoclastic methanogenesis, which is consistent with previous coalbed stimulation results (Jones et al., 2010). Furthermore, Robbins et al. (2016) suggested that the increase in methane production from lower rank coals is directly related to higher acetate concentrations, which is consistent with the results of this and other studies (McKay et al., 2022; Schweitzer et al., 2022).

Archaeal communities were found to be correlated with percent volatile matter, vitrinite reflectance, fixed carbon, maximum methane production rate, and the presence of algal amendment (Figure 7), indicating a dependence on coal rank, coal composition, and algal amendment. The significant correlation between archaeal community composition and the presence of algal amendment suggests that methanogens are more dependent on substrate availability than bacterial populations in these systems. Considering the enrichment of organisms from the bacterial family Pseudomonadaceae, the source of hydrogenotrophic methanogenesis substrates in amended treatments is likely from enhanced coal degradation. The enrichment of organisms previously shown to be capable of hydrogenotrophic methanogenesis is evidence that methane production in higher ranked coals can be stimulated, even with low *in-situ* acetate concentrations. Additionally, the limited abundance of acetoclastic methanogens in high rank coal microcosms may be explained by the high abundance of Geobacteraceae, who are known acetate scavengers when appropriate electron acceptors are available. It is possible that members of the family Geobacteraceae outcompete acetoclastic methanogens when acetate concentrations are low, providing another explanation for lower methane production in high rank coals.

## Conclusion

The research presented in this study aims to clarify the relationship between biogenic methane production and coal rank. It was found that methane production is generally higher in subbituminous coals than in thermally mature bituminous coal. Importantly, this study shows that significant biogenic methane yields are possible from coals that were previously thought to have limited bioavailability, and that previously reported methane yields from high rank coals were likely an under-estimate due to the lack of an active coal-degrading microbial community. Furthermore, this study affirms the assertion that archaeal community and methanogenesis pathways are sensitive to coal rank, with low rank coals promoting acetoclastic methanogenesis, as previously reported (Jones et al., 2010). This study re-examined the biogenic methane yield from previously investigated coals and found significantly higher methane yields, highlighting the importance of using coal-adapted, *in-situ* microbial consortia from coalbed methane sources (i.e., CBM reservoirs) in laboratory studies to accurately predict biogenic methane potential from coal. While matching the inoculum source with the methane producing coal source (i.e., the same coal seam) would be a further improvement to this study, the results presented here suggest that using any coal-derived inoculum can result in higher methane yields compared to studies that use non-coal derived inocula. Additionally, it was determined that the addition of algal amendment can enhance methane production rates and decrease lag times before the onset of methane production across a variety of coal ranks by shifting microbial communities towards known hydrocarbon degrading organisms that have previously been identified in coal environments. This result expands the potential viability of this stimulation technique to previously overlooked CBM resources. Further investigation is needed to describe the mechanisms of MECBM production in higher rank coals to ensure field-scale feasibility.

## Data availability statement

The datasets forming the basis for Figures 1, 2, 3, and S1, estimating the total amounts of methane and carbon dioxide detected in the headspace of the treatments are available at https://doi.org/10.5061/dryad.59zw3r2cn. The sequencing data presented in the study are deposited in NCBI’s Sequence Read Archive (SRA), with the accession number PRJNA938141.

## Author contributions

GP designed and performed experimental work, analyzed data, and wrote and revised manuscript. KD contributed to experimental design, the revision and contents of manuscript with comments and feedback. HDS and HJS performed sequencing for microbial community analysis and contributed to microbial data analysis and manuscript revisions. MF contributed to the revision of the manuscript with comments and feedback. EB contributed to experimental design, the revision of the manuscript with comments and feedback. RG contributed to experimental design, data analysis, the writing and revision of the manuscript with comments and feedback. All authors contributed to the article and approved the submitted version.

## Funding

Funding for this research was provided by the Department of Energy (DE-FE0024068) and by the United States Geological Survey (USGS) Energy Resources Program. Partial financial support was provided by the National Science Foundation (NSF) under grant #s CHE-1230632 and 1736255. Environmental samples used in this work were collected at a field site managed by USGS. Any opinions, findings, conclusions, or recommendations expressed herein are those of the authors and do not necessarily reflect the views of the DOE and NSF. Any use of trade, firm, or product names is for descriptive purposes only and does not imply endorsement by the US Government.

## Conflict of interest

The authors declare that the research was conducted in the absence of any commercial or financial relationships that could be construed as a potential conflict of interest.

## Publisher’s note

All claims expressed in this article are solely those of the authors and do not necessarily represent those of their affiliated organizations, or those of the publisher, the editors and the reviewers. Any product that may be evaluated in this article, or claim that may be made by its manufacturer, is not guaranteed or endorsed by the publisher.
