# Supplementary material for: Algal amendment enhances biogenic methane production from coals of different thermal maturity
Source: Front Microbiol. 2023 Mar 10;14:1097500. doi: 10.3389/fmicb.2023.1097500 (PMC10036379; doi:10.3389/fmicb.2023.1097500)
Supplement: Supplementary file 1 [file Table_1.DOCX]

Supplementary Material

# Supplementary Tables

Table S1: Archaeal species richness using Inverse Simpson Index of sequenced coal samples.

| Coal Sample | Number of Sequences | Coverage | Observed OTUs | Chao | Inverse Simpson |
| --- | --- | --- | --- | --- | --- |
| Lignite Algae | 32785 | 0.999603 | 165 | 167 | 9.5 |
| Lignite | 47278 | 0.99981 | 211 | 212 | 10.3 |
| SubC | 18778 | 0.999148 | 111 | 115 | 9.1 |
| SubB Algae | 404 | 0.987624 | 23 | 26 | 3.7 |
| SubB | 39756 | 0.999698 | 260 | 261 | 10.6 |
| HV Bit (Stockton) Algae | 59073 | 0.999865 | 196 | 197 | 8.7 |
| HV Bit (Stockton) | 14431 | 0.998961 | 105 | 109 | 9.9 |
| HV Bit (Pittsburgh) Algae | 7764 | 0.99781 | 68 | 78 | 6.0 |
| HV Bit (Pittsburgh) | 42878 | 0.999813 | 176 | 177 | 11.9 |
| LV Bit Algae | 10408 | 0.998751 | 69 | 75 | 5.5 |
| LV Bit | 26580 | 0.999586 | 161 | 162 | 8.3 |
| GB Algae | 28622 | 0.999441 | 190 | 192 | 12.4 |
| Sequencing Control | 4 | 0.75 | 2 | 2 | 2.0 |

Table S2: Bacterial species richness using Inverse Simpson Index of sequenced coal samples.

| Coal Sample | Number of Sequences | Coverage | Observed OTUs | Chao | Inverse Simpson |
| --- | --- | --- | --- | --- | --- |
| SubC Algae | 26970 | 0.999852 | 151 | 151 | 4.7 |
| SubC | 30070 | 0.999834 | 182 | 182 | 7.4 |
| SubB Algae | 20928 | 0.999857 | 45 | 46 | 3.0 |
| SubB | 20652 | 0.999806 | 83 | 83 | 2.3 |
| HV Bit (Stockton) Algae | 16921 | 0.999645 | 104 | 105 | 3.5 |
| HV Bit (Stockton) | 7973 | 0.999749 | 81 | 81 | 3.5 |
| HV Bit (Pittsburgh) Algae | 36917 | 0.999946 | 89 | 89 | 1.8 |
| HV Bit (Pittsburgh) | 7883 | 0.999239 | 83 | 84 | 5.5 |
| LV Bit Algae | 44695 | 0.999911 | 102 | 102 | 2.4 |
| LV Bit | 4520 | 0.999115 | 96 | 96 | 8.4 |
| GB Algae | 19763 | 0.999848 | 110 | 110 | 5.7 |
| Sequencing Control | 36851 | 1.000000 | 18 | 18 | 1.5 |

**Table S3:** Tukey Pairwise Comparisons from Generalized Linear Model comparing cumulative CH_4_ production (μmol CH_4_/g coal) considering amendment condition and coal rank. Means that do not share a letter are statistically significantly different.

| Treatment | N | Mean | Grouping | | | | | |
| --- | --- | --- | --- | --- | --- | --- | --- | --- |
| SubC Algae | 3 | 65.2883 | A |  |  |  |  |  |
| SubC | 3 | 59.1528 | A |  |  |  |  |  |
| SubB Algae | 3 | 45.1272 |  | B |  |  |  |  |
| Lignite Algae | 3 | 38.6500 |  | B | C |  |  |  |
| SubB | 3 | 35.7184 |  | B | C |  |  |  |
| HV Bit (Pittsburgh) Algae | 3 | 35.7121 |  | B | C |  |  |  |
| HV Bit (Stockton) | 3 | 32.3924 |  |  | C |  |  |  |
| HV Bit (Stockton) Algae | 3 | 31.8380 |  |  | C |  |  |  |
| Lignite | 3 | 31.5495 |  |  | C |  |  |  |
| LV Bit Algae | 3 | 26.9764 |  |  | C | D |  |  |
| HV Bit (Pittsburgh) | 3 | 26.9232 |  |  | C | D |  |  |
| LV Bit | 3 | 19.2581 |  |  |  | D | E |  |
| GB Algae | 3 | 11.7026 |  |  |  |  | E | F |
| GB | 3 | 0.0000 |  |  |  |  |  | F |

**Table S4:** Tukey pairwise comparisons from Generalized Linear Model comparing maximum methane production rate (μmol CH_4_/g coal/day) considering amendment condition and coal rank. Means that do not share a letter are statistically significantly different.

| Treatment | Sampling Interval | N | Mean | Grouping | | | | | |
| --- | --- | --- | --- | --- | --- | --- | --- | --- | --- |
| SubC Algae | Day 38-56 | 3 | 1.72108 | A |  |  |  |  |  |
| SubB Algae | Day 16-38 | 3 | 1.60735 | A | B |  |  |  |  |
| Lignite Algae | Day 16-38 | 3 | 1.29841 | A | B | C |  |  |  |
| SubC | Day 56-75 | 3 | 1.27764 | A | B | C |  |  |  |
| HV Bit (Pittsburgh) Algae | Day 16-38 | 3 | 1.06343 | A | B | C | D |  |  |
| SubB | Day 38-56 | 3 | 0.92906 |  | B | C | D | E |  |
| HV Bit (Stockton) Algae | Day 16-38 | 3 | 0.91194 |  | B | C | D | E |  |
| Lignite | Day 38-56 | 3 | 0.76619 |  |  | C | D | E |  |
| HV Bit (Stockton) | Day 16-38 | 3 | 0.67441 |  |  | C | D | E | F |
| HV Bit (Pittsburgh) | Day 38-56 | 3 | 0.64437 |  |  | C | D | E | F |
| LV Bit Algae | Day 38-56 | 3 | 0.59193 |  |  | C | D | E | F |
| GB Algae | Day 0-16 | 3 | 0.40550 |  |  |  | D | E | F |
| LV Bit | Day 75-92 | 3 | 0.28540 |  |  |  |  | E | F |
| GB | N/A | 3 | 0.00000 |  |  |  |  |  | F |

**Table S5:** Tukey pairwise comparisons from Generalized Linear Model comparing cumulative CO_2_ production (μmol CO_2_/g coal) considering amendment condition and coal rank. Means that do not share a letter are significantly different.

| Treatment | N | Mean | Grouping | | | | |
| --- | --- | --- | --- | --- | --- | --- | --- |
| SubC Algae | 3 | 75.8366 | A |  |  |  |  |
| SubC | 3 | 71.9528 | A |  |  |  |  |
| SubB Algae | 3 | 23.6338 |  | B |  |  |  |
| SubB | 3 | 15.1586 |  | B | C |  |  |
| HV Bit (Pittsburgh) Algae | 3 | 12.7888 |  | B | C | D |  |
| HV Bit (Stockton) Algae | 3 | 12.5944 |  | B | C | D |  |
| GB Algae | 3 | 7.8076 |  |  | C | D | E |
| HV Bit (Pittsburgh) Algae | 3 | 6.0129 |  |  | C | D | E |
| Lignite Algae | 3 | 4.6446 |  |  | C | D | E |
| LV Bit | 3 | 3.6965 |  |  | C | D | E |
| HV Bit (Stockton) | 3 | 3.5477 |  |  | C | D | E |
| LV Bit Algae | 3 | 2.5888 |  |  |  | D | E |
| Lignite | 3 | 1.1087 |  |  |  | D | E |
| GB | 3 | -3.1177 |  |  |  |  | E |

**Table S6:** Tukey Pairwise Comparisons from Generalized Linear Model comparing maximum CO_2_ production rate (μmol CO_2_/g coal/day) considering amendment condition and coal rank. Means that do not share a letter are significantly different.

| Treatment | Sampling Interval | N | Mean |  |  |  |  |  |
| --- | --- | --- | --- | --- | --- | --- | --- | --- |
| SubC Algae | Day 0-16 | 3 | 3.44847 |  | A |  |  |  |
| SubC | Day 0-16 | 3 | 3.27046 |  | A |  |  |  |
| SubB Algae | Day 0-16 | 3 | 1.11503 |  |  | B |  |  |
| SubB | Day 0-16 | 3 | 0.70846 |  |  | B | C |  |
| HV Bit (Pittsburgh) Algae | Day 0-16 | 3 | 0.61349 |  |  | B | C | D |
| HV Bit (Stockton) Algae | Day 0-16 | 3 | 0.60235 |  |  |  | C | D |
| GB Algae | Day 0-16 | 3 | 0.35883 |  |  |  | C | D |
| HV Bit (Pittsburgh) | Day 0-16 | 3 | 0.27709 |  |  |  | C | D |
| Lignite Algae | Day 38-56 | 3 | 0.19060 |  |  |  |  | D |
| GB | Day 38-56 | 3 | 0.17007 |  |  |  |  | D |
| Lignite | Day 38-56 | 3 | 0.16133 |  |  |  |  | D |
| LV Bit | Day 56-75 | 3 | 0.15500 |  |  |  |  | D |
| HV Bit (Stockton) | Day 0-16 | 3 | 0.15483 |  |  |  |  | D |
| LV Bit Algae | Day 56-75 | 3 | 0.12373 |  |  |  |  | D |
|  |  |  |  |  |  |  |  |  |

**Table S7:** Methane yield in headspace through 60 days of incubation. Percent difference is calculated as the difference between the observed methane yield and the observed methane yield in Fallgren et al., 2013 for the same coal samples.

| Coal Rank | Platt et al. (this study)  (μmol CH_4_/g coal) | Fallgren et al. (2013)  (μmol CH_4_/g coal) | Percent Difference |
| --- | --- | --- | --- |
| Lignite | 29.39 | 0.24 | 99.2 |
| SubC | 24.40 | - | - |
| SubB | 34.27 | 0.38 | 98.9 |
| HV Bit (Stockton) | 26.28 | - | - |
| HV Bit (Pittsburgh) | 20.53 | 1.41 | 93.1 |
| LV Bit | 8.72 | 2.47 | 71.7 |

# Supplementary Figures


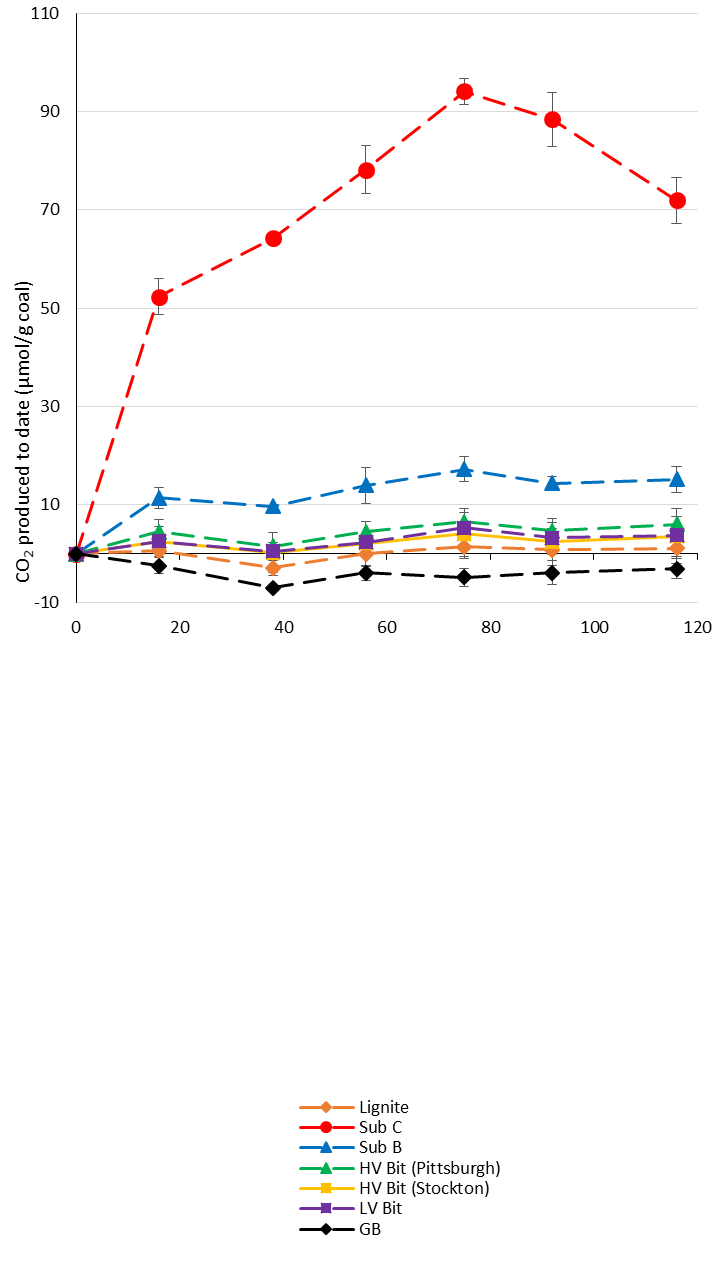

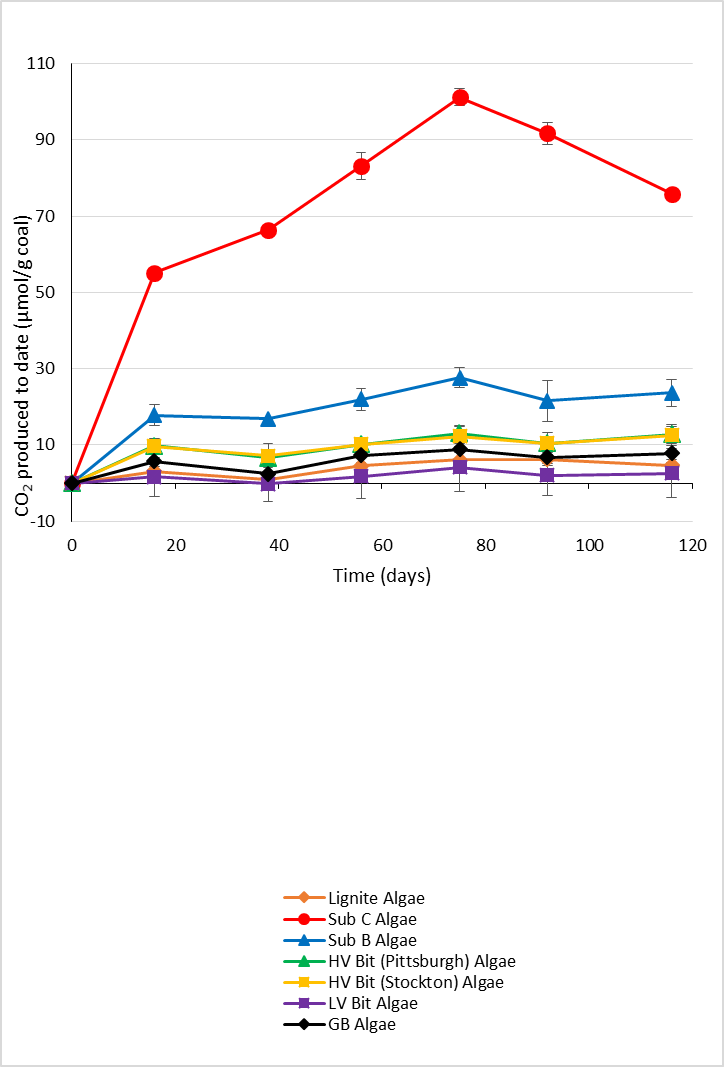
­­­­
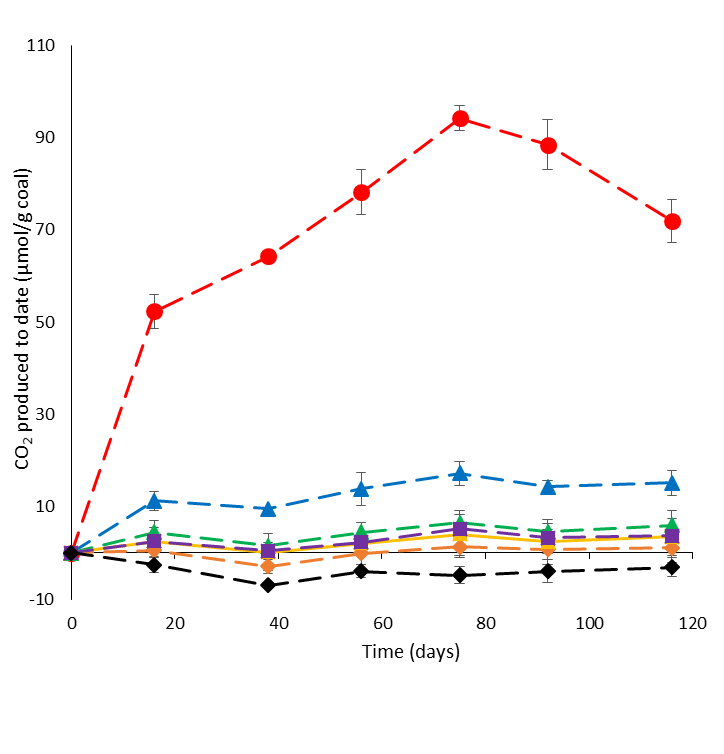

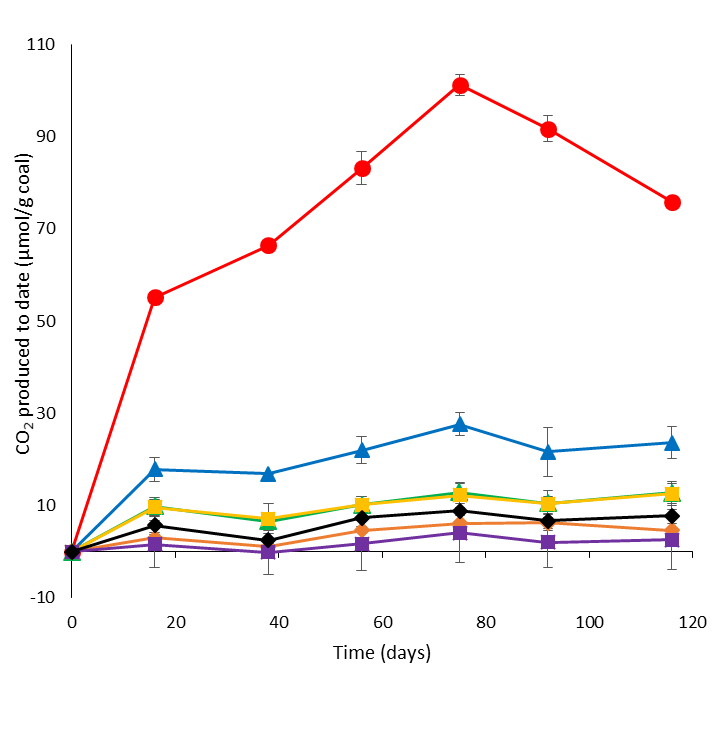


Figure S1: Carbon dioxide produced for SLA-04 algae extract amended microcosms (top) and unamended microcosms (bottom). For amended microcosms algae extract was added to a final concentration of 0.1 g/L. Error bars represent one standard deviation.
